# Molecular model of the outward facing state of the human P-glycoprotein (ABCB1), and comparison to a model of the human MRP5 (ABCC5)

**DOI:** 10.1186/1742-4682-4-33

**Published:** 2007-09-06

**Authors:** Aina W Ravna, Ingebrigt Sylte, Georg Sager

**Affiliations:** 1 Department of Pharmacology, Institute of Medical Biology, University of Tromsø, N-9037 Tromsø, Norway

## Abstract

**Background:**

Multidrug resistance is a particular limitation to cancer chemotherapy, antibiotic treatment and HIV medication. The ABC (ATP binding cassette) transporters human P-glycoprotein (ABCB1) and the human MRP5 (ABCC5) are involved in multidrug resistance.

**Results:**

In order to elucidate structural and molecular concepts of multidrug resistance, we have constructed a molecular model of the ATP-bound outward facing conformation of the human multidrug resistance protein ABCB1 using the Sav1866 crystal structure as a template, and compared the ABCB1 model with a previous ABCC5 model. The electrostatic potential surface (EPS) of the ABCB1 substrate translocation chamber, which transports cationic amphiphilic and lipophilic substrates, was neutral with negative and weakly positive areas. In contrast, EPS of the ABCC5 substrate translocation chamber, which transports organic anions, was generally positive. Positive-negative ratios of amino acids in the TMDs of ABCB1 and ABCC5 were also analyzed, and the positive-negative ratio of charged amino acids was higher in the ABCC5 TMDs than in the ABCB1 TMDs. In the ABCB1 model residues Leu65 (transmembrane helix 1 (TMH1)), Ile306 (TMH5), Ile340 (TMH6) and Phe343 (TMH6) may form a binding site, and this is in accordance with previous site directed mutagenesis studies.

**Conclusion:**

The Sav1866 X-ray structure may serve as a suitable template for the ABCB1 model, as it did with ABCC5. The EPS in the substrate translocation chambers and the positive-negative ratio of charged amino acids were in accordance with the transport of cationic amphiphilic and lipophilic substrates by ABCB1, and the transport of organic anions by ABCC5.

## Background

The transport of small organic molecules and ions across cell membranes generally requires a transporter protein, and these transporter proteins have recognition sites that make them specific for particular substrates. Drugs can interact with these recognition sites and inhibit the transporter, or be substrates themselves. There is an increasing focus on transporters as drug targets, and the information on transporter structure and function is rapidly increasing. The number of drugs interacting with transporters will probably increase in the future.

According to the transporter classification approved by the transporter nomenclature panel of the International Union of Biochemistry and Molecular Biology [1,2], transporters are divided into classes based on both function and phylogeny. These classes are: 1. Channels and pores, 2. Electrochemical potential-driven transporters (secondary transporters), 3. Primary active transporters, 4. Group translocators, 5. Transport electron carriers, 8. Accessory factors involved in transport, 9. Incompletely characterized transport systems.

ABC (ATP binding cassette) transporters belong to class 3 (primary active transporters), subclass A (diphosphate bond hydrolysis-driven transporters) and family 1 (ABC superfamily) [1]. Primary active transporters use a primary source of energy to drive active transport of particles from regions of low concentration to regions of high concentration. The ABC superfamily transporters are structurally related membrane proteins sharing a common intracellular motif that exhibits ATPase activity that cleaves ATP's terminal phosphate, using the free energy from ATP (adenosine triphosphate) stored in the high-energy phosphate bond as the energy source for activating the transporter [1-4].

The human genome encodes more than 40 ABC transporters divided into five different subfamilies: ABCA, ABCB, ABCC, ABCD and ABCG, based on phylogenetic analysis (Additional file 1). According to the TCDB [1], these subfamilies belong to subclasses 3.A.1.201–212, ABC-type efflux permeases (mostly eukaryotic) [2]. The ABC genes are highly conserved between species, indicating that most of these genes have been present since the beginning of eukaryotic evolution [5]. These transporters feature both transmembrane domains (TMD) and nucleotide binding domains (NBD). In general, the domain arrangement of these transporters is TMD-NBD-TMD-NBD, but TMD0-TMD-NBD-TMD-NBD, NBD-TMD-NBD-TMD, TMD-NBD and NBD-TMD also exist [5,6]. TMD0 is a 5 TMH amino-terminal domain present in ABCC1, ABCC2 and ABCC3. The NBD contains the Walker A and B motifs [7] and a signature C motif. Two further subfamilies, ABCE and ABCF, are related to ABC transporters, but they lack transmembrane domains and thus are not membrane transporters [4,5]. The substrate specificity is provided by the TMDs, which contain 6–11 transmembrane helices (TMHs) [5].

Cells exposed to toxic compounds can develop resistance by a number of mechanisms, including increased excretion. The result is multidrug resistance (MDR), which is a particular limitation to cancer chemotherapy, antibiotic treatment and HIV medication. Transporters in subfamilies ABCA, ABCB, ABCC and ABCG are involved in multidrug resistance [8-11]. Development of inhibitors of drug efflux transporters has been sought for use as supplement to therapy to overcome multidrug resistance [12]. In order to elucidate structural and molecular concepts of multidrug resistance, we have focused on the TMDs of ABCB1 and ABCC5 using molecular modeling techniques. ABCB1 and ABCC5 both have a TMD-NBD-TMD-NBD arrangement, with TMDs consisting of 6 TMHs. ABCB1 transports cationic amphiphilic and lipophilic substrates [13-16], while ABCC5 transports organic anions [17,18]. Information about the molecular aspects of ligand interactions with these transporters can be used to design therapeutic agents that may aid to overcome multidrug resistance.

Several electron density maps of ABCB1 have been published [19-22], giving insight into ABCB1 architecture. The latest electron density map had a resolution limit of ~8 Å, and although this structure reveals the TMH packing of ABCB1, it is not possible, at this resolution, to predict the TMH numbering [22]. In lack of an X-ray crystal structure, molecular modeling by homology may be an alternative for gaining structural insight into protein drug targets. The bacterial ABC transporter Sav1866 from *Staphylococcus aureus *has been crystallized in an outward-facing ATP-bound state [23]. Sav1866 is a bacterial homologue to ABCB1 [23], indicating that the Sav1866 crystal structure could be used as a template for the present model building by homology. The 12 TMH arrangement of the Sav1866 crystal structure is consistent with the electron density maps of ABCB1 [23]. The NBDs of both Sav1866 and ABCB1 are functionally equivalent; both NBDs are responsible for ATP binding and hydrolysis [23,24].

In this study we have constructed an ABCB1 model based on the Sav1866 crystal structure [23] using molecular modeling techniques. Among the transporters in the ABCC subfamily (multidrug resistance proteins, MRPs), ABCC5 has a "P-gp-like" ("ABCB1-like") core domain organization (TMD1-NBD1-TMD2-NDB2) [25]. We have previously constructed an ABCC5 model in a cGMP docking study (submitted) using the Sav1866 crystal structure [23] as a template, and in the present study we have performed a comparative analysis of the ABCB1 and ABCC5 models in order to understand the molecular concepts of the substrate difference between ABCB1 and ABCC5. The comparative analysis included the electrostatic potential surfaces (EPS) of the substrate translocation chambers, and the positive-negative ratios of charged amino acids of the TMDs of both models. A phylogenetic analysis of human ABC transporters has been performed in order to understand the phylogenetic relationship between ABCB1 and ABCC5. The ABCB1 model has been compared with cross-linking and site directed mutagenesis data published on ABCB1 [26-32].

## Results

### Evolutionary tree of the human ABC transporters

**Figure 1:**
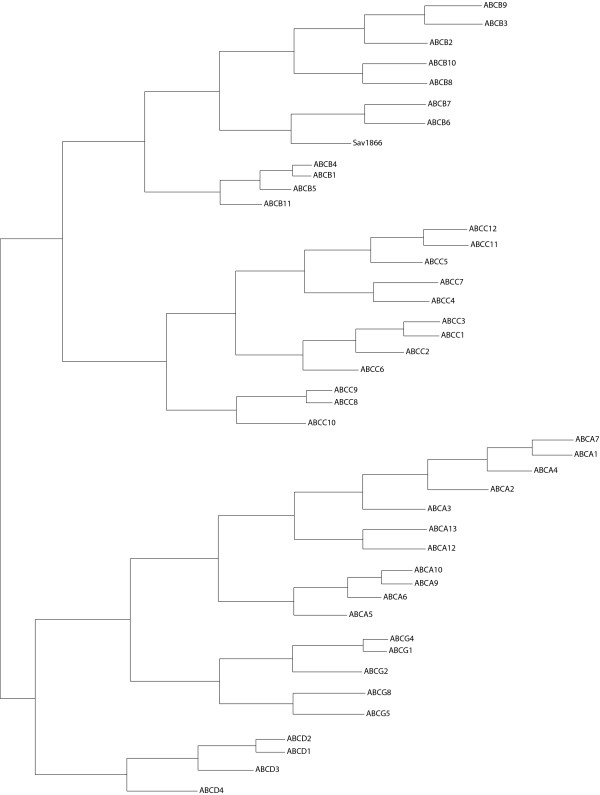
**Evolutionary tree**. Evolutionary tree of the human ABC efflux permeases, together with Sav1866. The topmost branch (the "ABCB-branch") includes ABCB1 and Sav1866, while the next branch (the "ABCC-branch") includes ABCC5.

The evolutionary tree of the human ABC transporters, together with Sav1866, is shown in Figure 1. ABCB1 and Sav1866 are localized on the same branch of the evolutionary tree (the "ABCB-branch"), while ABCC5 is localized on a different branch (the "ABCC-branch").

### Amino acid sequence identities of TMDs and positive-negative ratios of charged amino acids

Table 1 shows the amino acid sequence identities between the Sav1866-TMD, ABCB1-TMD1, ABCB1-TMD2, ABCC5-TMD1, and ABCC5-TMD2. The TMD with the highest sequence identity with the Sav1866 TMD is the ABCC5-TMD1 (21 %), while the TMD with the lowest sequence identity with the Sav1866 TMD is the ABCC5-TMD2 (16 %). Both ABCB1 TMDs share a 17 % sequence identity with Sav1866. The percentages (%) of the charged amino acids aspartate (D), glutamate (E), histidine (H), lysine (K), and arginine (R), and positive-negative ratios of amino acids in the ABCB1 and ABCC5 TMDs are shown in Table 2. While the positive-negative ratio of amino acids is 1.1 (1.4 when histidine is included) in the ABCB1 TMDs, the corresponding ratio in the ABCC5 TMDs is 1.5 (1.8 when histidine is included). Thus the positive-negative ratio of amino acids is higher in the ABCC5 TMDs than in the ABCB1 TMDs. The charged amino acids were mainly localized in the substrate translocation chamber.

**Table 1:** Amino acid sequence identities. The amino acid sequence identities (%) between Sav1866-TMD, ABCB1-TMD1, ABCB1-TMD2, ABCC5-TMD1, and ABCC5-TMD2.

**TMDs**	**Sav1866-TMD**	**ABCB1-TMD1**	**ABCB1-TMD2**	**ABCC5-TMD1**	**ABCC5-TMD2**
**Sav1866 -TMD**	100	17	17	21	16
**ABCB1-TMD1**		100	30	16	21
**ABCB1-TMD2**			100	21	19
**ABCC5-TMD1**				100	15
**ABCC5-TMD2**					100

**Table 2:** Positive-negative ratios of charged amino acids.

	**Start-end**	**D%**	**E%**	**H%**	**K%**	**R%**	**D+E%**	**K+R%**	**H+K+R%**	(K+R)(D+E) MathType@MTEF@5@5@+=feaafiart1ev1aaatCvAUfKttLearuWrP9MDH5MBPbIqV92AaeXatLxBI9gBaebbnrfifHhDYfgasaacH8akY=wiFfYdH8Gipec8Eeeu0xXdbba9frFj0=OqFfea0dXdd9vqai=hGuQ8kuc9pgc9s8qqaq=dirpe0xb9q8qiLsFr0=vr0=vr0dc8meaabaqaciaacaGaaeqabaqabeGadaaakeaadaWcaaqaaiabcIcaOiabbUealjabgUcaRiabbkfasjabcMcaPaqaaiabcIcaOiabbseaejabgUcaRiabbweafjabcMcaPaaaaaa@364C@	(H+K+R)(D+E) MathType@MTEF@5@5@+=feaafiart1ev1aaatCvAUfKttLearuWrP9MDH5MBPbIqV92AaeXatLxBI9gBaebbnrfifHhDYfgasaacH8akY=wiFfYdH8Gipec8Eeeu0xXdbba9frFj0=OqFfea0dXdd9vqai=hGuQ8kuc9pgc9s8qqaq=dirpe0xb9q8qiLsFr0=vr0=vr0dc8meaabaqaciaacaGaaeqabaqabeGadaaakeaadaWcaaqaaiabcIcaOiabbIeaijabgUcaRiabbUealjabgUcaRiabbkfasjabcMcaPaqaaiabcIcaOiabbseaejabgUcaRiabbweafjabcMcaPaaaaaa@3845@
**ABCC5-TMD1**	179 – 454	1.1	4.3	0.7	5.4	4.3	5.4	9.7	10.4	1.7	1.8
**ABCC5-TMD2**	848–1147	3.7	2.3	2.3	2.7	5.3	6	8	10.3	1.3	1.7
**ABCC5, both TMDs**										1.5	1.8
**ABCB1-TMD1**	52–346	3.7	5.1	1.4	4.7	3.4	8.8	8.1	9.5	0.9	1.1
**ABCB1-TMD2**	711–994	2.8	3.5	1.1	4.9	4.2	6.3	9.1	10.2	1.4	1.6
**ABCB1, both TMDs**										1.1	1.4

Positive-negative ratios of charged amino acids. The percentages % of aspartate (D), glutamate (E), histidine (H), lysine (K), and arginine (R), and positive-negative ratios of charged amino acids in ABCB1-TMD1, ABCB1-TMD2, ABCC5-TMD1, and ABCC5-TMD2. The positive-negative ratio of amino acids is higher in the ABCC5 TMDs (1.5, 1.8 including histidine) than in the ABCB1 TMDs (1.1, 1.4 including histidine).

### ABCB1 model

**Figure 2:**
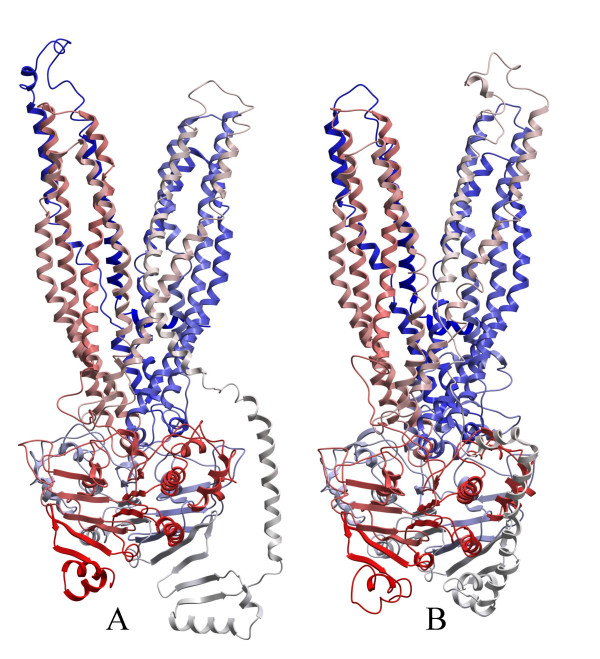
**ABCB1 and ABCC5 models**. C_α _traces of the ABCB1 (Panel A) and ABCC5 (Panel B) models viewed in the membrane plane, with the extracellular side facing upwards. Color code of the models is blue via white to red from N-terminal to C-terminal.

The refined ABCB1 and ABCC5 (submitted) models are shown in Figure 2, panels A and B. The loop connecting NBD1 and TMD2 of ABCB1 was mainly α-helical from residues 623–703, except from a parallel β-sheet formed between residues 614–618 and residues 646–650, and an extended stretch from residues 651–657. The first part of this loop was folded and covering NBD1 of ABCB1 towards the cytoplasm. A central cavity perpendicular to the cell membrane was formed by TMD1 and TMD2, and TMHs 1, 2, 3, 5, 6, 7, 8, 9, 11 and 12 contributed to the cavity lining. TMH5 and TMH2 of TMD1 were packed against TMH8 and TMH11 of TMD2, respectively, with mainly hydrophobic interactions. The substrate translocation chamber was closed towards the intracellular side, and the TMDs were twisted relative to the NBDs. The TMHs diverged into two symmetrical parts towards the extracellular side, one part consisting of TMHs 1 and 2 of TMD1 and TMHs 9–12 of TMD2, and one part consisting of TMHs 7 and 8 of TMD2 and TMHs 3–6 of TMD1 (Figure 2). Interactions between the NBDs were relatively hydrophilic, and the secondary structure of the areas of each NBD forming the contact area between the two NBDs was generally in extended conformation. The NBDs, having the same fold as the NBDs of the Sav1866 crystal structure, were tightly packed at the intracellular side of the membrane, containing the nucleotide binding sites formed by the motifs Walker A, Walker B, Q-loop and switch regions.

### EPS of the substrate translocation chamber

**Figure 3:**
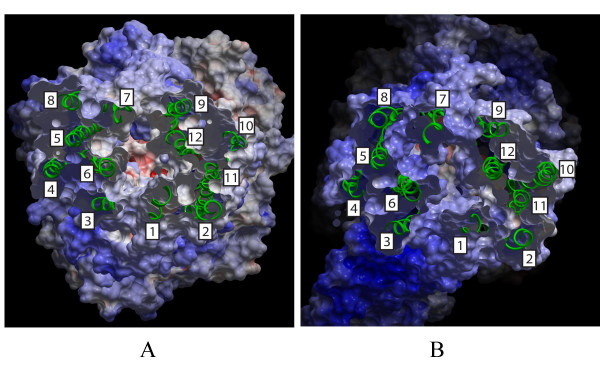
**Electrostatic potentials surface (EPS)**. The electrostatic potentials surface (EPS) of the substrate translocation chambers of ABCB1 (Panel A) and ABCC5 (Panel B) viewed from the intracellular side with blue areas indicating positive areas and red areas indicating negative areas. TMHs are displayed as green ribbons. TMH numbering is indicated in white boxes.

Figure 3 shows the EPS of the substrate translocation chambers of ABCB1 (Panel A) and ABCC5 (Panel B). While the EPS of the substrate translocation chamber of ABCB1 was neutral with negative and weakly positive areas, the EPS of the ABCC5 substrate translocation chamber was generally positive.

### Quality validation

The overall quality factor of ABCB1, as shown by the Errat option of the Savs Metaserver, was 96.2, and a value above 90 indicates a good model. According to the Ramachandran plot provided by the Procheck option, 87.1 % of the ABCB1 residues were in the most favored regions, 11.7 % were in additional allowed regions, 0.8 % were in generously allowed regions, and 0.4 % were in disallowed regions. The summary of the Whatcheck option reported that the model was satisfactory.

## Discussion

Several ABCB1 models have previously been published [33-36] based on MsbA X-ray crystal structures that later were retracted [37]. The 12 TMHs of the present ABCB1 model are arranged as the TMHs of the Sav1866 crystal structure [23], and both are consistent with the electron density maps of ABCB1 [22].

As shown in Additional file 1, the human ABC efflux transporters comprise a large group of transporters featuring a wide range of functions and selectivities. ABC efflux transporters play important roles in physiological processes by transporting ligands such as bile salts/acids, conjugated steroids, cyclic nucleotides, ions, heme, lipids, antigens, retinoids, peptides, leukotrienes, organic anions, cations, and cholesterol, and many of them are involved in drug efflux.

Since ABCB1 transports cationic amphiphilic and lipophilic substrates [13-16] and ABCC5 transports organic anions [17,18], the substrate translocation chamber localized in the TMDs of these transporters were of particular interest from a pharmacological point of view. The EPS of the substrate translocation chamber of ABCB1 was neutral with negative and weakly positive spots (Figure 3A). In contrast, the substrate translocation chamber of ABCC5 was generally positive (Figure 3B). An amino acid charge difference could also be seen between the TMDs of the two transporters (Table 2), with a lower positive-negative amino acid ratio of ABCB1 than of ABCC5. Thus, ABCB1, which transports cationic amphiphilic and lipophilic substrates, has a more neutral substrate translocation chamber than ABCC5, which has a positive chamber that transports organic anions. Substrates for these transporters bind to a binding site accessible to the intracellular side of the transporters. During the translocation process the binding site changes conformation, and the substrates are released to the extracellular side. The Sav1866 structure is captured in an outward facing conformation with the pore representing an extrusion pocket, rather than a binding pocket, and the modeled ABCB1 pore also represents an extrusion pocket. Even though the conformation changes, from a high affinity binding site (substrate recognition) to a low affinity binding site (substrate extrusion), the amino acids in the translocation area will be expected to contribute to similar ESP in both conformations.

Several cross-linking and site directed mutagenesis data have been published on ABCB1 [26-32]. These studies have indicated that TMH6 and TMH12 may take part in ligand binding [26,27,30,31]. Cross-linking has also shown that TMH5 and TMH8 are near each other [28], and that TMH2 and TMH11 are near each other [29]. As shown in Figure 3A, the present ABCB1 model is consistent with these experimental data; TMH6/TMH12, TMH5/TMH8 and TMH2/TMH11 are indeed adjacent. Comparing the reported residues from the experimental studies with the orientations of these residues in the present ABCB1 model verifies that the pore-lining residues of the TMHs are correctly localized, confirming that the alignment used for the ICM modeling procedure is realistic. Cross-linking studies have shown that residue pairs Asn266-Gly774, Ile299-Phe770, Ile299-Gly774, and Gly300-Phe770 (TMH5 and TMH8, respectively), are adjacent [28]. In the present ABCB1 model, these residue pairs are in direct contact with each other. According to cross-linking studies, Val133 and Cys137 (TMH2) are close to Ala935 and Gly939 (TMH11) [29], and this is also in accordance with the ABCB1 model. Furthermore, experimental studies have suggested that Leu65 (TMH1) [31], Ile306 (TMH5) [32], Ile340 (TMH6) [26,31], Phe343 (TMH6) [27], Phe728 (TMH7) [32], and Val982 (TMH12) [30] may participate in ligand binding. All these residues line the aqueous pore of the ABCB1 model and may indeed have ligand contact.

**Figure 4:**
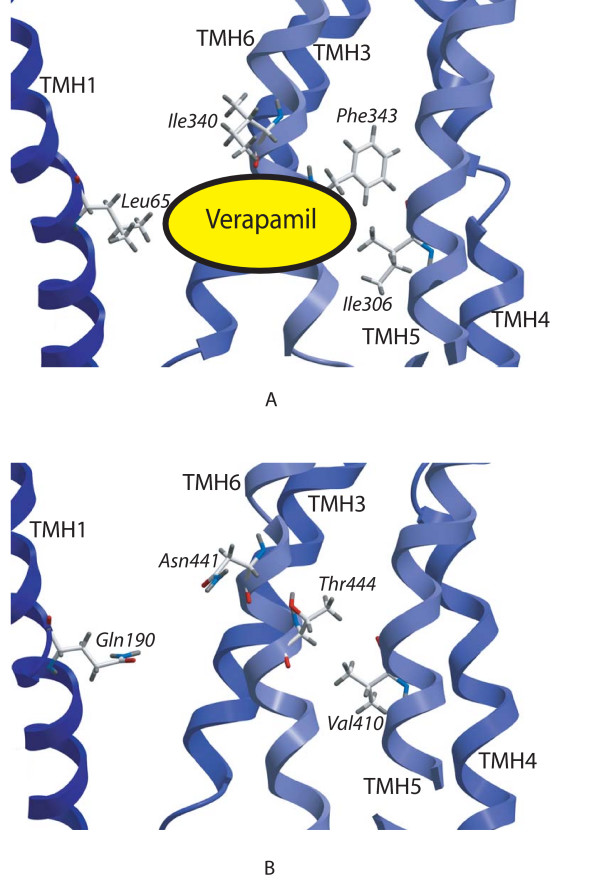
**Ligand interaction areas**. Close-up of putative ligand interaction areas of ABCB1 (Panel A) and ABCC5 (Panel B). The view is a cross-section of the transporters perpendicular to the membrane. The oval shaped object with the text "Verapamil" (Panel A) indicates where Verapamil binding may take place. TMHs are shown as blue C_α _traces. Color coding of displayed residues: Carbon: White; Hydrogen: Grey; Oxygen: Red; Nitrogen: Blue. Panel A: Residues Leu65 (TMH1) [30], Ile306 (TMH5) [30], Ile340 (TMH6) [25, 30] and Phe343 (TMH6) [26] have been shown to interact with ligands in site directed mutagenesis studies. Panel B: Corresponding residues in ABCC5 are Gln190 (TMH1), Val410 (TMH5), Asn441 (TMH6) and Thr444 (TMH6) respectively.

Site directed mutagenesis studies on ABCB1 have proposed a verapamil binding site including residues Leu65 (TMH1) [31], Ile306 (TMH5) [31], Ile340 (TMH6) [26,31] and Phe343 (TMH6) [27]. In the ABCB1 model these residues may form a binding site (Figure 4A). Ligand interactions between the TMH6 residues Ile340 and Phe343 and rhodamine have also been proposed in an ABCB1 modeling and docking study [33]. The corresponding residues in ABCC5 are Gln190 (TMH1), Val410 (TMH5), Asn441 (TMH6) and Thr444 (TMH6), respectively (Figure 4B). Gln190 (TMH1), Asn441 (TMH6) and Thr444 (TMH6) of ABCC5 have previously been proposed to take part in ligand binding in a previous MRP5 modeling and cGMP docking study (submitted). Interestingly, the above mentioned ABCB1 residues are more lipophilic than the corresponding ABCC5 residues. This is in accordance with the lipophilic efflux featured by ABCB1, and with the more neutral EPS of the ABCB1 substrate translocation chambers.

Even though the EPS differences between the substrate translocation chambers of ABCB1 and ABCC5 are in accordance with their substrate specificity differences, one can not be certain that the Sav1866 crystal structure is a suitable template. According to the evolutionary tree (Figure 1), there are five main clusters of ABC efflux transporters: ABCA, ABCB, ABCC, ABCD and ABCG. Two main branches are seen, with ABCB, ABCC and ABCD in one branch, and ABCA and ABCG in the other branch. ABCB and ABCC subfamilies are closer related to each other than to the ABCD subfamily. Sav1866 is situated on the "ABCB-branch". The evolutionary tree thus indicates that an Sav1866 crystal structure may be a suitable template for at least ABCB1. The phylogeny of ABC transporters is based on homology of their NBDs [2], which is why the number of TMHs may differ within one subfamily, such as in subfamily ABCC5, where ABCC1 has 17 TMHs and ABCC5 has 12 TMHs. Even though ABCB1 and ABCC5 are localized on different branches in the evolutionary tree, they both have a common core domain organisation (TMD1-NBD1-TMD2-NDB2) [25]. The identity between Sav1866 and ABCB1 is 31%. Accurate predictions can be made with an amino acid sequence similarity greater than 50 % between the target and the template protein, but even with very low homologies there may be considerable structural similarities, such as for the G-protein coupled receptors and bacteriorhodopsin, where the sequence similarities within the transmembrane regions are 6–11% [38]. The conservation of the secondary structure elements is also relevant, since active sites and functional domains can have very similar geometries, even for distantly related proteins. The sequence identity between Sav1866 and ABCC5 is 23%, and phylogenetic analyses of ABC transporters have indicated that eukaryotic ABCB transporters (including ABCB1), ABCC transporters (including ABCC5), and bacterial ABC transporters have a common ancestor, and that they have similar domain organizations [39]. Among the ABCC transporters, ABCC5 is most similar to ABCB1 [40], indicating that the Sav1866 X-ray crystal structure could also be used as a template for constructing an ABCC5 model by homology. The identity between the Sav1866-TMD and the ABCC5-TMD1 is actually higher (21%) than the identity between the Sav1866-TMD and the ABCB1-TMD1 (17%). In comparison, the sequence identity between the human serotonin transporter (SERT) and the crystal structure of the bacterial homologue from *Aquifex aeolicus *(LeuT_Aa_) is ~20%, and several SERT models have been made been made using the LeuT_Aa _as a template [41,42]. The TMD sequence identities between the Sav1866-TMD and the ABCB1- and ABCC5-TMDs thus indicate that they have an overall similar organization and that the Sav1866-TMD may have been a suitable template for modeling the TMD segments of ABCB1 and ABCC5.

Membrane proteins may be highly flexible, metastable molecules, making them generally difficult to crystallize, and it has been suggested for the major facilitator transporter *Escherichia coli *lactose permease symporter (Lac Permease) that substrate binding in transporters may result in widespread conformational changes, and scissors like movements and sliding or tilting motions may occur during turnover [43]. The crystal structure of Sav1866 indicates that domain swapping and subunit twisting takes place in the transport cycle [23]. Thus, the substrate may be "pumped" from the inside of the membrane, binding with high affinity to the binding site, to the outside of the membrane, binding with low affinity, and thus being expelled to the extracellular space [44]. It is therefore possible that the Sav1866 crystal structure represents a substrate expelling state where the binding site has changed drastically into a low affinity conformation through twisting and squeezing movements.

The calculations did not include water molecules or membrane phospholipids, and this omission may have influenced the model structure. The N- and C-terminals and two loops of ABCB1, the loop connecting TMH1 and TMH2, and the loop connecting NBD1 and TMD2, are relatively long and are not accounted for in the Sav1866 crystal structure. These segments are outside the limits for reliable loop generation via PDB searches and could not be predicted or modeled with confidence. Thus, The N- and C-terminals were not included in the model, but the two loops were included in order to get a more correct distribution of masses and electrostatics in the calculations than in a model with gaps were these loops are. Anyhow, it should be kept in mind that loops of such lengths modeled with computational techniques for loop modeling are relatively inaccurate, and, consequently, these were the most uncertain parts of the model. The fragment-based ab initio ROSETTA approach to the prediction of protein structure [45] may have been used, but the conformations of the modeled loops would still be too uncertain because of their lengths. Thus, the most certain regions of the ABCB1 model are the NBDs, because of their high level of sequence identity to the NBDs of Sav1866, and the TMD parts, which are in accordance with cross-linking and site directed mutagenesis data published on ABCB1 [26-32], confirming that porelining residues of the TMHs are correctly localized. The most uncertain parts are the loop connecting TMH1 and TMH2, and the loop connecting NBD1 and TMD2, which implies that these regions should only be considered as relatively crude approximations. Since the loop connecting NBD1 and TMD2 started 17 amino acids further towards the N-terminal, the NBD1 region had amino acids in its C-terminal end that was modeled as a loop instead of with homology to the NDB1 of Sav1866. Thus, the conformation of this 17 amino acid segment is uncertain, but this short segment does not include the Walker A and B motifs and is not a major part of NBD1. The loops are probably highly flexible, so any conformation generated by molecular modeling will only be a model of a temporary loop conformation. Anyhow, since the substrate binding area is of particular interest from a pharmacological point of view, focus was kept on the TMH area, and not the loops, in this molecular modeling study.

## Conclusion

Making crystals of membrane proteins is in general technically difficult, and when no X-ray crystal structure is available, molecular modeling is a step forward towards structural knowledge of drug targets such as ABCB1 and ABCC5. In this study, the molecular concepts of the substrate specificity differences between ABCB1 and ABCC5 have been visualized using molecular modeling techniques. Even though there are uncertainties concerning the overall models, it seems that both site directed mutagenesis data [26,27,31] and the EPS in the substrate translocation chambers are in accordance with the transport of cationic amphiphilic and lipophilic substrates by ABCB1 [13-16], and the transport of organic anions by ABCC5 [17,18]. This, and the consistency with the latest electron density map of ABCB1 [22], indicates that the Sav1866 X-ray structure [23] may serve as a suitable template for the ABCB1 and ABCC5 models. The ABCB1 model presented here is considered as a working tool to aid experimental studies. Eventually, membrane transport modulating agents may be developed, which may be used in the search for overcoming multidrug resistance.

Co-ordinates of the ABCB1 and ABCC5 models are available from the authors upon request.

## Methods

### Phylogenetic analysis of human ABC transporters

The Swiss-Prot Protein knowledgebase [46] and the TCDB [2] were used to retrieve fasta files of human ABC transporters, together with their Swiss-Prot accession codes, their synonyms and TCDB classification numbers. The ICM software version 3.4–4 [47] was used to create a multiple sequence alignment and an evolutionary tree of the human ABC transporters, together with Sav1866. The ICM software creates evolutionary trees by the neighbor-joining method [48].

The amino acid sequence identities between the TMDs of Sav1866, ABCB1 and ABCC5 were retrieved using the ICM software. The start and endpoints of the TMDs were 14–298 (Sav1866), 177–451 (ABCC5, TMD1), 852–1147 (ABCC5, TMD2), 52–350 (ABCB1, TMD1), and 709–992 (ABCB1, TMD2). Positive-negative ratios of amino acids in the TMDs of ABCB1 and ABCC5 were also analyzed using the ICM software.

### Homology modeling of ABCB1

**Figure 5:**
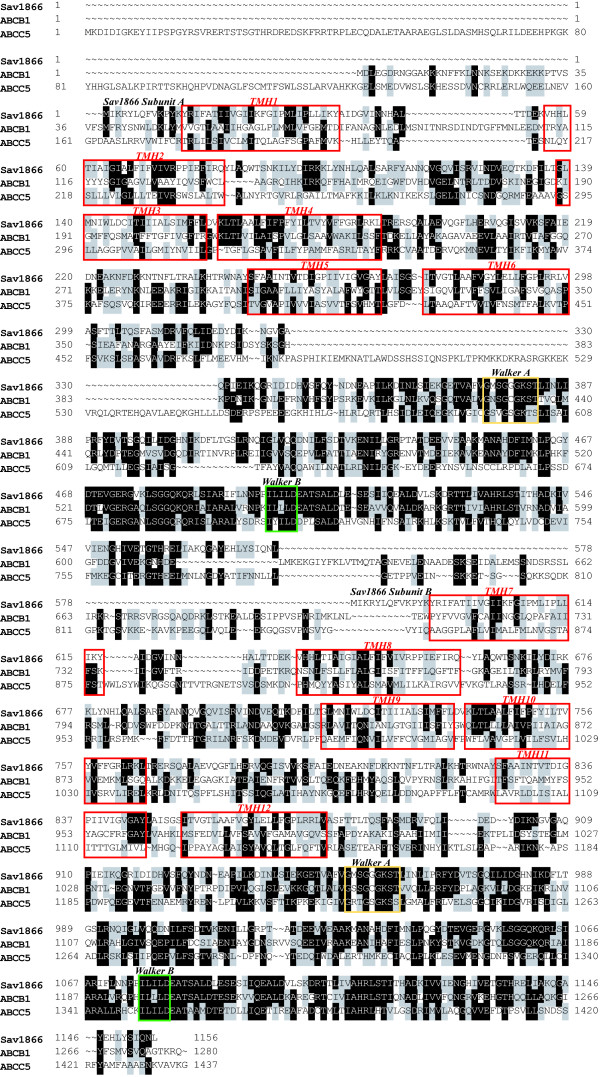
**Alignment**. Alignment of VC-Sav1866, ABCB1 and ABCC5 used as input alignment for the ICM homology modeling module. TMHs are indicated in red boxes, Walker A motifs are indicated in yellow boxes, and Walker B motifs are indicated in green boxes.

The crystal structure of Sav1866 [23] (pdb code 2HYD), which has a 3 Å resolution, was used as template to construct a homology model of ABCB1 (Swiss-Prot accession code P08183), using the ICM software versions 3.4–9b [47]. T-COFFEE, Version 4.71 available at the Le Centre national de la recherche scientifiquewebsite [49], and ICM version 3.4–4 [47], were used to create multiple sequence alignments of human ABCB1, human ABCC5, human ABCC11 (SWISS-PROT accession number Q9BX80), human ABCC4 (SWISS-PROT accession number O15439), Sav1866 (SWISS-PROT accession number Q99T13), *Vibrio cholerae *MsbA (SWISS-PROT accession number Q9KQW9) and *Escherichia coli *MsbA (SWISS-PROT accession number P60752). The alignments were used as a basis, and adjusted in ICM for gaps for the input alignment in the ICM homology modeling module. To strengthen the sequence alignment, secondary structure predictions were performed to define the boundaries of the TMHs using the PredictProtein server for sequence analysis and structure prediction [50], and SWISS-PROT [46]. The alignment of Sav1866, ABCB1 and ABCC5 is shown in Figure 5. The ICM homology modeling module constructs the model from a few core sections defined by the average of C_α _atom positions in the conserved regions. Loops are constructed by searching within thousands of high quality structures in the PDB databank [51] by matching them in regard to sequence similarity and sterical interactions with the surroundings of the model. The best fitting loops are selected based on their relative energies. N- and C-terminals were not included in the models. Because of the length of the loop connecting NBD1 and TMD2, the loop was particularly difficult to model. In the generated models of the loop, the residues had a tendency to overlap with surrounding amino acids (sterical clashes), and more than 20 models was constructed before a model without sterical clashes was generated. In order to accomplish this, the start of the loop was moved one amino acid further towards the N-terminal direction, or the end of the loop was moved one amino acid further towards the C-terminal direction, in the ICM input alignment per modeling round, making the input loop longer until a model with no sterical clashes was generated. The alignment shown in figure 5 is the exact input alignment used for the final model. The construction of the ABCC5 model is described in a previous cGMP docking study (submitted).

### Model refinement

Globally optimizing of the side-chain positions and annealing of the backbones were performed with the RefineModel macro of ICM. This macro first performs a side-chain conformational sampling using "Montecarlo fast" [52], a program module that samples conformational space of a molecule with the ICM global optimization procedure. Iterations of the procedure consist of a random move followed by a local energy minimization, followed by a complete energy calculation. Based on the energy and the temperature, the iteration is accepted or rejected. After the "Montecarlo fast" module, an iterative annealing of the backbone with tethers provided is performed. These tethers are harmonic restraints pulling an atom in the model to a static point in space represented by a corresponding atom in the template. Finally a second Monte Carlo side-chain sampling is performed. ECEPP3 charges [53] were used for the amino acids, and a surface based implicit solvation model [47] was included in the calculations.

The ABCB1 model was subjected to two subsequent energy minimizations by the AMBER 8.0 program package, using the leaprc.ff03 force field [54]. The first energy minimization was performed with restrained backbone by 500 cycles of steepest descent minimization followed by 500 steps of conjugate gradient minimization, and the second energy minimization was performed with no restraints by 1000 cycles of steepest descent minimization followed by 1500 steps of conjugate gradient minimization. A 10 Å cut-off radius for nonbonded interactions and a dielectric multiplicative constant of 1.0 for the electrostatic interactions were used in these minimizations. Membrane molecules were not included in the model refinements. The electrostatic potential surface (EPS) of the ABCB1 model was calculated with the ICM program, with a potential scale from -10 to +10.

### Quality validation of the ABCB1 model

The stereochemical quality of the ABCB1 model was checked using the Savs Metaserver for analyzing and validating protein structures [55]. Programs run were Procheck [56], What_check [57], and Errat [58].

## Competing interests

The author(s) declare that they have no competing interests.

## Authors' contributions

AWR carried out the molecular modeling studies (homology modeling and model refinement, and quality validation), created sequence alignments and the evolutionary tree, and drafted the manuscript. IS participated in the design of the study, and contributed with bioinformatics advice and critical review of the manuscript. GS conceived of the study and participated in its design, contributed with biological advice and critical review of the manuscript. All authors read and approved the final manuscript.

## Supplementary Material

Additional file 1 Table S1. A list of human ABC-type efflux transporters, with their Swiss-Prot accession codes, synonyms and TCDB classification numbers. Click here for file
