# Supplementary material for: Molecular model of the outward facing state of the human P-glycoprotein (ABCB1), and comparison to a model of the human MRP5 (ABCC5)
Source: Theor Biol Med Model. 2007 Sep 6;4:33. doi: 10.1186/1742-4682-4-33 (PMC2211457; doi:10.1186/1742-4682-4-33)
Supplement: Additional file 1 — Table S1. A list of human ABC-type efflux transporters, with their Swiss-Prot accession codes, synonyms and TCDB classification numbers. [file 1742-4682-4-33-S1.doc]

Table S1 Human ABC-type efflux transporters, with **their** Swiss-Prot accession codes, **synonyms and TCDB classification numbers.**

| **Sub-family** | **Member** | **Acc. Number (SwissProt)** | **Synonyms (Swissprot//TCDB)** | **TCDB Classification number** |
| --- | --- | --- | --- | --- |
| ABCA | ABCA1 | O95477 | ABC-1, Cholesterol efflux regulatory protein, Cholesterol/phospholipid flippase | 3.A.1.211.1 |
|  | ABCA2 | Q9BZC7 | ABC2, Multidrug resistance pump | 3.A.1.211.3 |
|  | ABCA3 | Q99758 | ABC-C | Not present |
|  | ABCA4 | P78363 | RIM ABC transporter, RIM protein, RmP, Stargardt disease protein, The retinal-specific ABC transporter | 3.A.1.211.2 |
|  | ABCA5 | Q8WWZ7 | None | Not present |
|  | ABCA6 | Q8N139 | None | Not present |
|  | ABCA7 | Q9BZC4 | None | Not present |
|  | ABCA9 | Q8IUA7 | None | Not present |
|  | ABCA10 | Q7Z2I9 | None | Not present |
|  | ABCA12 | Q86UK0 | ATP-binding cassette 12 | Not present |
|  | ABCA13 | Q86UQ4 | None | Not present |
| ABCB | ABCB1 | P08183 | Multidrug resistance protein 1 (MDR1), P-glycoprotein 1, Broad specificity multidrug resistance (MDR) efflux pump | 3.A.1.201.1 |
|  | ABCB2 | Q03518 | APT1, Peptide transporter TAP1, Peptide transporter PSF1, Peptide supply factor 1, PSF-1, MHC heterodimeric peptide exporter | 3.A.1.209.1 |
|  | ABCB3 | Q03519 | APT2, Peptide transporter TAP2, Peptide transporter PSF2, Peptide supply factor 2, PSF-2, MHC heterodimeric peptide exporter | 3.A.1.209.1 |
|  | ABCB4 | P21439 | Multidrug resistance protein 3 (MDR3), P-glycoprotein 3, Short chain fatty acid phosphatidylcholine translocase | 3.A.1.201.3 |
|  | ABCB5 | Q6KG50 | P-glycoprotein | Not present |
|  | ABCB6 | Q9NP58 | Mitochondrial ABC transporter 3, ABC transporter umat, Iron transporter | 3.A.1.210.6 |
|  | ABCB7 | O75027 | ABC transporter 7 protein (mitochondrial), ABC7 iron transporter, (X-linked sideroblastis anemia protein) | 3.A.1.210.4 |
|  | ABCB8 | Q9NUT2 | Mitochondrial ATP-binding cassette 1, M-ABC1 | Not present |
|  | ABCB9 | Q9NP78 | TAP-like protein, TAPL, hABCB9 | Not present |
|  | ABCB10 | Q9NRK6 | Mitochondrial ATP-binding cassette 2, M-ABC2 | Not present |
|  | ABCB11 | O95342 | Bile salt export pump, BSEP or SPGP | 3.A.1.201.2 |
| ABCC | ABCC1 | P33527 | MRP1, Leukotriene C(4) transporter, LTC4 transporter, Drug resistance pump | 3.A.1.208.8 |
|  | ABCC2 | Q92887 | Canalicular multispecific organic anion transporter 1, MRP2, Canalicular multidrug resistance protein, Hepatic canalicular conjugate exporter | 3.A.1.208.2 |
|  | ABCC3 | O15438 | Canalicular multispecific organic anion transporter 2, MRP3, Multi-specific organic anion transporter-D, MOAT-D | 3.A.1.208.9 |
|  | ABCC4 | O15439 | MRP4, Multi-specific organic anion transporter-B, MOAT-B | 3.A.1.208.7 |
|  | ABCC5 | O15440 | MRP5, Multi-specific organic anion transporter-C, MOAT-C, pABC11, SMRP | Not present |
|  | ABCC6 | O95255 | MRP6, Anthracycline resistance-associated protein, Multi-specific organic anion transporter-E, MOAT-E, Multidrug (anthracycline) resistance organic anion efflux pump, The pseudoxanthoma elasticum disease protein) | 3.A.1.208.10 |
|  | ABCC7 | P13569 | Cystic fibrosis transmembrane conductance regulator, CFTR, cAMP-dependent chloride channel | 3.A.1.202.1 |
|  | ABCC8 | Q09428 | Sulfonylurea receptor 1, SUR1 | 3.A.1.208.4 |
|  | ABCC9 | O60706 | Sulfonylurea receptor 2, SUR2 | Not present |
|  | ABCC10 | Q5T3U5 | MRP10 | Not present |
|  | ABCC11 | Q96J66 | MRP8, Cyclic nucleotide (cAMP and cGMP) efflux pump | 3.A.1.208.13 |
|  | ABCC12 | Q96J65 | None | Not present |
| ABCD | ABCD1 | P33897 | Adrenoleukodystrophy protein, ALDP, Long chain fatty acid (LCFA) transporter | 3.A.1.203.3 |
|  | ABCD2 | Q9UBJ2 | Adrenoleukodystrophy-related protein., hALDR, Adrenoleukodystrophy-like 1 | Not present |
|  | ABCD3 | P28288 | 70 kDa peroxisomal membrane protein, PMP70, Peroxysomal long chain fatty acyl (LCFA) transporter | 3.A.1.203.1 |
|  | ABCD4 | O14678 | Peroxisomal membrane protein 69, PMP69, Peroxisomal membrane protein 1-like, PXMP1-L, P70R | Not present |
| ABCG | ABCG1 | P45844 | White protein homolog, ATP-binding cassette transporter 8 | Not present |
|  | ABCG2 | Q9UNQ0 | Placenta-specific ABC transporter, Breast cancer resistance protein, Mitoxantrone resistance-associated protein, CDw338 antigen | 3.A.1.204.2 |
|  | ABCG4 | Q9H172 | None | Not present |
|  | ABCG5 | Q9H222 | Sterolin-1 | 3.A.1.204.5 |
|  | ABCG8 | Q9H221 | Sterolin-2 | 3.A.1.204.5 |
